# Granulomatous Hepatitis Following Intra-Vesical Instillation of Bacillus Calmette–Guérin for Treatment of Bladder Cancer

**DOI:** 10.3390/idr13030057

**Published:** 2021-07-01

**Authors:** Elsa Alves Branco, Raquel Duro, Teresa Brito, António Sarmento

**Affiliations:** 1 Infectious Diseases Department, Centro Hospitalar e Universitário de São João, 4099-002 Porto, Portugal; raquel.duro@gmail.com (R.D.); antonio.sarmento@chsj.min-saude.pt (A.S.); 2 Internal Medicine Department, Centro Hospitalar e Universitário de São João, 4099-002 Porto, Portugal; mtapbrito@gmail.com

**Keywords:** granulomatous hepatitis, bacillus Calmette–Guérin, bladder cancer

## Abstract

Intra-vesical instillation of bacillus Calmette–Guérin (BCG) is an important treatment modality of superficial bladder cancer. It is usually well tolerated, although some adverse reactions can occur. One possible yet rare complication is granulomatous hepatitis, that is thought to be caused either by BCG infection or a hypersensitivity reaction to the bacillus. We present a case of a 79-year-old apparently immunocompetent patient who developed granulomatous hepatitis a few months after BCG administration for bladder cancer immunotherapy. It is important to notice that acid-fast smears and cultures are often negative, and these should not exclude diagnosis nor delay treatment. Our case highlights the importance of clinical suspicion and prompt initiation of appropriate treatment.

## 1. Introduction

Bladder instillations with bacillus Calmette–Guérin (BCG), an attenuated live strain of *Mycobacterium bovis*, are commonly used as immunotherapy for bladder carcinoma [1,2]. The mechanism by which BCG exerts its antitumour activity is unknown, but it has been suggested that a non-specific immune response to BCG might also destroy tumour cells. Another suggested mechanism is that the severe inflammation caused by BCG leads to local ischaemia, thereby killing tumour cells [1].

Although usually well tolerated, both local and systemic BCG-related complications may occur following instillation, with early (in a few hours) or late (in several months) presentation [2,3]. Local side effects are frequent (90%), and may include urinary frequency, cystitis, fever and haematuria [4,5]. Although serious complications are rare, patients can develop severe, life-threatening sepsis with disseminated mycobacterial infection [5]. Various other local and systemic side effects have been reported, such as acute respiratory failure and septic shock, isolated renal tuberculosis and granulomatous hepatitis [4].

Granulomatous hepatitis is a rare serious side effect, which has been considered a hypersensitivity reaction to BCG or a BCG infection [1]. Steg et al. and Lamm et al. described a 3% and 0.7% incidence, respectively, of BCG-related granulomatous hepatitis after intra-vesical instillation [4,6,7]. Hepatitis usually presents with fever, anorexia, jaundice and alteration of liver function tests, typically with a cholestatic pattern of serum liver tests; liver biopsy reveals granulomas in all cases [2]. The pathogenic mechanisms underlying the development of these complications remain not fully understood, and considerable debate exists about whether it represents a form of hypersensitivity reaction, based on the histologic finding of granulomas in the absence of recoverable microorganisms, or an active mycobacterial infection, since some authors have demonstrated viable bacilli in a variety of tissues [2].

## 2. Case Presentation

We present a case of a 79-year-old male patient, with history of dyslipidaemia medicated with statins, moderate alcohol consumption (half a bottle of red wine a day) and ischaemic heart failure. He was diagnosed with high-grade papillary urothelial carcinoma in 2016 and was submitted to immunotherapy with intra-vesical instillation of BCG for a full year (last instillation in February 2018).

**Figure 1:**
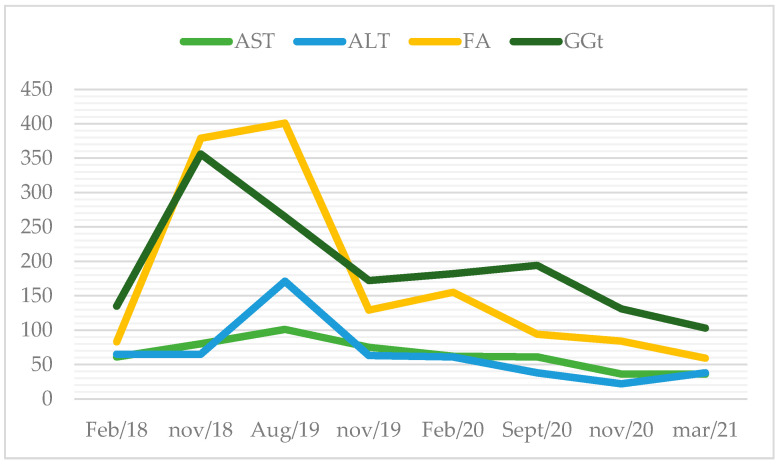
Evolution of liver enzymes over the years (in U/L).

Routine bloodwork documented hepatic cytolysis and cholestasis for the first time in January of 2018 (aspartate transaminase (AST) 74 U/L (normal range: 10–37 U/L); alanine transaminase (ALT) 55 U/L (normal range: 10–37 U/L), gamma-glutamyl transferase (GGT) 273 U/L (normal range: 10–49 U/L); alkaline phosphatase (AP) 176 U/L (normal range: 30–120 U/L)), without hyperbilirubinaemia. Figure 1 describes the evolution of liver enzymes. He complained of fatigue and diminished appetite. On physical examination, the liver was palpable 4 cm bellow the right costal margin. Blood tests showed mild hepatic cytolysis. Three months later, he had aggravated liver enzymes, with AST and ALT up to 2.5 x/upper normal limit and cholestasis of 3–7 x/UNL with normal bilirubin. At that time, he stopped statin therapy and alcohol consumption. 

**Figure 2:**
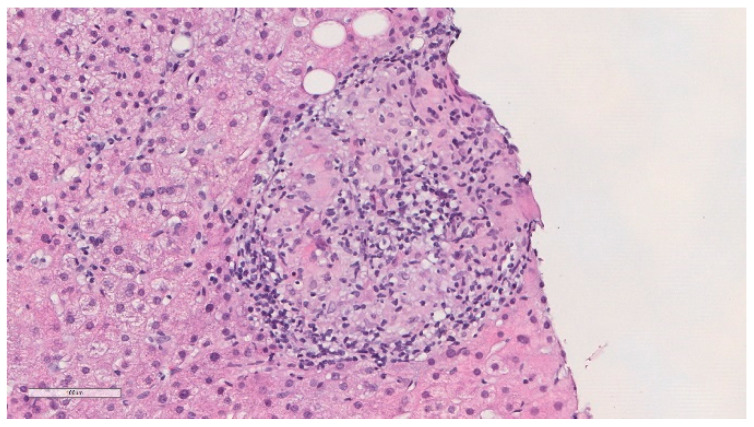
Histopathological findings of the liver biopsy sample revealed epithelioid granulomas (haematoxylin and eosin).

He was previously evaluated at outpatient internal medicine appointments. An abdominal ultrasound performed in November 2018 showed a globous hepatic parenchyma with no focal lesions. In April of 2019, he complained about weight loss (about 4 kg) in the previous few months and colangio-magnetic resonance was performed that showed homogeneous mild hepatomegaly and splenomegaly, without nodules or other lesions, and the absence of biliary duct dilatation. In November 2019, he was electively admitted to undergo a hepatic biopsy. Histology showed granulomatous hepatitis and fibrotic septation of the parenchyma, with some multinucleated cells but without the typical aspects of Langhans cells and negative Ziehl–Neelsen stain (Figure 2). PCR of Mycobacterium tuberculosis complex was negative in the liver biopsy paraffined specimen. HIV and viral hepatitis serologies were all negative. Serology and polymerase chain reaction (PCR) for Coxiella burnetii and Brucella spp., as well as mycobacterial examination of urine, were also negative. Thoracic computed tomography was performed and revealed multiple mediastinal and hilar lymph nodes without adenomegaly criteria, some with gross calcifications, and lung parenchyma with evident emphysema, without masses, nodules or consolidations. An immune panel for autoimmune hepatitis was normal, and the serum angiotensin conversion enzyme (ACE) was negative.

The patient was then referred to the infectious diseases outpatient clinic, where he was first observed in June 2020. Given the existence of a granulomatous hepatitis in a patient previously submitted to BCG intra-vesical instillation, with accompanying complaints of anorexia and weight loss, the diagnosis of granulomatous hepatitis caused by BCG infection was postulated. The hepatic cytolysis and cholestasis started 1–2 months after discontinuation of the intra-vesical immunotherapy, which was consistent with the suspected diagnosis. Empirical treatment with isoniazid 300 mg/day and rifampicin 600 mg/day was started in late June. After treatment initiation, there was a progressive improvement of the liver enzymes, full normalisation of the cytolysis in November and a considerable decrease in cholestasis. The patient received 9 months of dual anti-bacillary treatment, with complete recovery, and was discharged in March 2021.

## 3. Discussion

Disseminated BCG disease is a rare but life-threatening complication of BCG administration [5]. The adverse effects of intra-vesical BCG instillations can appear early or several years after the treatment [8]. The spectrum of symptoms may be similar to that of tuberculosis infection, including persistent fever, night sweats and weight loss [5]. There is still controversy relating to whether the clinical presentation of BCG systemic disease is caused by actual dissemination of *M. bovis* in the involved tissues or is secondary to a hypersensitivity reaction [3]. Noticeably, in the majority of cases, hypersensitivity and infection cannot be differentiated histopathologically and clinically [4].

BCG-related hepatitis is a rare complication, and most authors suggest that it is caused by a hypersensitivity reaction to BCG, based on negative Ziehl–Neelsen staining and negative cultures of liver tissue [1]. BCG-related granulomatous hepatitis should be considered in cases of abnormal liver function tests and persistent fever following BCG therapy [4]. Table 1, Table 2, and Table 3 summarise some reported cases of granulomatous hepatitis following intra-vesical instillation of BCG published in the literature over the last 3 decades. Liver biopsy reveals granulomas in all hepatitis cases, and a direct smear for mycobacteria plays an important role. However, the detection of acid-fast bacilli in liver, blood and bone marrow specimens is quite difficult. Even though acid-fast bacilli are positive in 10% of all liver tuberculosis biopsy samples, DNA hybridisation studies are often negative [4]. The exclusion of other entities and the prompt response to antituberculous treatment should be considered the cornerstones of diagnosis of BCG infection since cultures often remain negative [2]. In cases with an appropriate clinical presentation, negative culture tests should not be a cause for treatment delay. It should be remembered that early treatment improves the chance of success [4]. Treatment should consist of isoniazid (300 mg daily) and rifampicin (600 mg daily) with or without ethambutol and/or corticosteroids [1,5,8]. Treatment with pyrazinamide is not recommended, as all forms of *M. bovis* are resistant. Clinical response is monitored by the decline and normalisation of liver function tests [4]. In the case of our patient, he had a consistent clinical scenario and positive histology, although a direct smear for acid-fast bacilli and PCR of the biopsy specimen were both negative. Although no samples were sent to culture, prompt favourable response to treatment further corroborated the diagnosis. Overall, the prognosis of BCG infection is good [2]. Our case is an illustrative example of how time-consuming and laborious the diagnosis of granulomatous hepatitis following BCG administration can be if clinical suspicion is low, highlighting the need for high clinical suspicion in patients with an appropriate clinical scenario who were previously treated with intra-vesical BCG, in order to promptly initiate appropriate treatment.

## Figures and Tables

**Table 1:** Summary of some cases of granulomatous hepatitis following intra-vesical BCG therapy published in the literature.

Publication	No. Cases	Sex	Age	Time Interval Between BCG Instillation and Diagnosis	Isolation of *M. bovis* on Culture	Molecular Detection of *M. bovis*	Acid-Fast Staining on Liver Biopsy	Therapy	Outcome
Hillel Y. Marans and Huseyin M. Bekirov; Granulomatous Hepatitis following intravesical Bacillus Calmette-Guerin therapy for bladder Carcinoma; The Journal of Urology, 1987; 0022-~34 7 /8'7 /1871-0111$02.00/0	1	M	62 y	2 y	No	No	Negative	300 mg isoniazid + 600 mg rifampin (duration unknown)	Resolution
Dino A. Graziano et al; A case of granulomatous hepatitis after intravesical Bacillus Calmette-Guerin administration; The Journal of Urology, 1991; 0022-5347/91/1464-1118$03.00/0	1	M	61 y	39 days	No	Yes (bone marrow)	Negative	300 mg isoniazid + 600 mg rifampin + 40 mg prednisone (duration unknown)	Resolution
Proctor DD, Chopra S, Rubenstein SC, Jokela JA, Uhl L. Mycobacteremia and granulomatous hepatitis following initial intravesical bacillus Calmette-Guerin instillation for bladder carcinoma. Am J Gastroenterol. 1993 Jul;88(7):1112-5. PMID: 8317415.	1	M	72 y	8 days	Yes (blood)	No	Positive (rare)	Ethambutol (2 months) + isoniazid and rifampin (12 months)	Not mentioned
Arzt MR, Forouhar F. Granulomatous hepatitis as a complication of intravesical Bacillus Calmette-Guerin therapy for bladder carcinoma. Ann Clin Lab Sci. 1995 Sep-Oct;25(5):409-13. PMID: 7486816	1	M	74 y	2 months	No	No	Negative	300 mg isoniazid + 600 mg rifampin (6 months)	Resolution
FWG Leebeek et al. Granulomatous hepatitis caused by Bacillus Calmette-Guerin (BCG) infection after BCG bladder instillation. Gut 1996;38: 616-618	1	M	69 y	14 days	No	Yes (liver biopsy)	Negative	Isoniazid + rifampin (duration unknown)	Resolution

**Table 2:** Summary of some cases of granulomatous hepatitis following intra-vesical BCG therapy published in the literature.

Publication	No. Cases	Sex	Age	Time Interval Between BCG Instillation and Diagnosis	Isolation of M. bovis on Culture	Molecular Detection of M. bovis	Acid-Fast Staining on Liver Biopsy	Therapy	Outcome
Robert E. Scully, Eugene J. Mark, William F. McNeely, Sally H. Ebeling; Weekly Clinicopathological Exercises. Case Records of the Massachusetts General Hospital. 1998. Volume 339, Number 12	1	M	57 y	6 weeks	Yes (blood)	No	Negative	Ofloxacin + ethambutol + amikacin (6 months) + 60 mg prednisone (tapered for 3 months)	Recurrence
B. Ozbakkaloglu et al. Granulomatous Hepatitis Following Intravesical Bacillus Calmette-Guerin Therapy. International Urology and Nephrology; 1999.31(1), pp.49-53	1	M	46 y	1 week	No	No	Negative	300 mg isoniazid + 600 mg rifampin (6 months)	Resolution
Steven M. Van Outryve et al. Bacillus Calmette–Guerin-induced granulomatous hepatitisin a patient with a superficial bladder carcinoma. European Journal of Gastroenterology & Hepatology 2004, 16:1027–1032	1	M	71 y	26 days	No	No	Negative	300 mg isoniazid + 600 mg rifampin (12 months) + 32 mg prednisone (tapered for 6 months)	Resolution
Aliye Soylu et al. Peritoneal tuberculosis and granulomatous hepatitis secondary to treatment of bladder cancer with Bacillus Calmette-Guerin. Annals of Clinical Microbiology and Antimicrobials 2009:8:12 doi: 10.1186/1476-0711-8-12	1	M	46 y	Not specified	No	No	Negative	Isoniazid + rifampin + ethambutol (6 months) + 60 mg methylprednisolone (tapered for 1,5 months)	Resolution

**Table 3:** Summary of some cases of granulomatous hepatitis following intra-vesical BCG therapy published in the literature.

Publication	No. Cases	Sex	Age	Time Interval Between BCG Instillation and Diagnosis	Isolation of M. bovis on Culture	Molecular Detection of M. bovis	Acid-Fast Staining on Liver Biopsy	Therapy	Outcome
Maddalena Adami et al. Granulomatous hepatitis after intravesical bacillus Calmette – Guérin treatment. Scandinavian Journal of Infectious Diseases, 2011; 43: 55–57	1	M	78 y	6 weeks	No	No	Negative	Isoniazid + rifampin + ethambutol (6 months)	Resolution
Y Eso, A Takai, S Arasawa, Y Ueda and H Seno. Hepatobiliary and Pancreatic: Granulomatous hepatitis due to disseminated bacillus Calmette–Guérin disease. Journal of Gastroenterology and Hepatology 32 (2017) 1538. doi:10.1111/jgh.13831	1	M	66 y	17 days	No	No	Negative	Isoniazid + rifampin + ethambutol (6 months)	Resolution
M. Moussa, M. Abou Chakra. Granulomatous hepatitis caused by Bacillus Calmette-Guerin (BCG) infection after BCG bladder instillation: a case report. Urology Case Reports 2018. http://doi.org/10.1016/j.eucr.2018.05.012, accessed on 1 July 2021	1	M	52 y	7 days	No	Yes (liver biopsy)	Negative	Isoniazid + rifampin + ethambutol (6 months)	Resolution
